# Abstracts from German Journals

**Published:** 1872-02

**Authors:** Ch. Raushenberg

**Affiliations:** Atlanta, Georgia


### ABSTRACTS FROM GERMAN JOURNALS.

BY CH. RAUSHENBERG, M.D., ATLANTA, GEORGIA.

*Croup and Diphtheritis. By Dr. Franz Hartman, at Wiesbaden. (Virchow's Archives, February,* 1871.)

All differences between these two diseases, which have so far been made, are partly based upon the anatomical changes, partly on the clinical development peculiar to these two processes. Three different opinions in relation to their nature have gained ground amongst the profession. Some consider croup and diphtheritis as one and the same process; others consider diphtheritis a higher grade of the croupous process; and a third party separates the two diseases as essentially different and non-identical. The representatives of this latter view look upon diphtheritis as a disease of the blood, which localizes itself in the larynx.

If the differences in the clinical course of these two pathological processes were sufficient for a discussion as to their identity or non-identity, it would furnish a justification for their separation as different diseases. It is not the object of this essay to enter into a demonstration of the causes by which these differences in the clinical development of the two processes is brought about, but to explain how the anatomical changes upon which they depend account successively for these differences. It must be remembered that one and the same process can give a different anatomical image, according to the organ in which it develops itself. Structural differences and duration may materially change the pathologico-anatomical image, even if the cardinal symptoms remain the same.

Both of these diseases are *exudative processes*, and the tissues involved are the mucous membranes of the pharynx and larynx.

Hartman explains that not only the capillaries, but also the lymphatics, are instrumental in producing the exudation, and enters minutely into the pathological anatomy of that exudation on and within mucous membranes. He holds that the capillaries are not the last extremities of the blood-vessels, but that they connect with extremely minute tubes, which carry the nutrient material within the tissues. These tubes are, under normal conditions, not intended, and fit to carry blood-corpuscles; but being elastic, they will, when distended by the afflux of an abnormally large quantity of fluid from the capillaries, circulate blood-corpuscles also, and assume the appearance of newly-formed capillaries. This fact accounts for the sudden appearance of new vascular formations in irritated mucous membranes, so often observed. He calls these tubes vasa serosa, as in a normal condition they carry only serum, no corpuscles.

He further confesses to the opinion, that *the lymphatics*, at their extreme ends, are open vessels, which do not reach the epithelial layer of mucous membranes, but communicate with the surface of these membranes by interstitial cavities in their tissue, which have been discovered and described, under different names, by Keber, Letzerich, Brücke, and Ludwig. There are also exists a direct connection of the capillaries with the lymphatics through the above-mentioned vasa serosa of Hartman, which are identical to the plasmatic circulatory system of Koelliker—a fact the discovery of which our author ascribes to Leydig.

Thus the nutrient material, the plasma, reaches through the capillaries and plasmatic system to the tissues, and is afterward reabsorbed by the extreme ends of the lymphatics, to be returned again to the general circulation. Hence, we will find the number and distribution of capillary lymphatics corresponding in the tissues to that of the blood-capillaries.

*The anatomical structure of mucous membranes admits, therefore, as we have seen, the possibility of plasma reaching their surface.* If this does not occur under normal conditions, it is only owing to the circumstance, that the capillaries furnish no more fluid than the lymphatics can reabsorb and carry off, leaving the aspiratory power of the veins out of consideration. Under abnormal circumstances, however, matters change, and plasma can exude and reach the surface. Thus we find an increased quantity of lymph, in every inflammation of the mucous membrane, at the inflamed spot, which is caused, not only by an increased supply of plasma by the capillaries, but also by a decreased resorption of the same by the lymphatics, in consequence of their compression, according to Loesch's investigations.* If this surplus of lymph does not appear on the surface of the membrane, it only proves that its increased quantity alone is not sufficient to produce this effect, and that other influences must coöperate with this condition to drive the lymph to the surface and produce a visible exudation—*a false membrane.*

This momentum Hartman finds in the strong muscular apparatus underlying the mucous membrane, forming the walls of the pharynx and partly of the larynx. The mucous membrane adheres to the surface of these muscles more or less firmly, follows its different motions, and, accordingly, is more or less firmly compressed by these motions. If this action of the muscles is strong enough, and the quantity of exuded fluid within the interstices of the mucous membrane large enough, the surplus lymph of the inflamed mucous membrane is driven to the surface, where it coagulates, either at the spot of exudation or a little below, as the declivity of the surface where it exudes may induce a slight downward movement of the fluid before coagulation can be accomplished.

Observation has taught that exudation in croup takes place at intervals, particularly during the infantile age; thus we have repeated crops of exudation.

The second exudation, in penetrating the tissues from within towards the surface, must necessarily meet with obstacles in its passage: first, by the obstruction of the orifices of the mucous membrane, caused by the first exudation and its coagulation; and, secondly, by the diminution of the power of the muscular contractions, which keeps pace with the increase of the inflammation, so that a time arrives when the power of the pharyngeal muscles is no longer sufficient to squeeze the exuded fluid from within to the surface.

This gives us, first, a coagulated layer of exudation on the external surface of the mucous membrane—croup; and, in

---

*Beiträge Zudem Verhalten der Lymphgefässe bei der Entzündung. Virchow's Archiv., Vol. xliv., No. 4. (Translated: Contributions to the Action of the Lymphatics in Inflammation.)

second order, an exudation remaining in the interstices of the mucous membrane—diphtheritis. Croup, therefore, always precedes diphtheritis.

Whenever the motions of the pharyngeal muscles, at the beginning of the process of exudation, are considerably lessened or suspended by pain from excessive inflammation, neither croup nor diphtheritis appears, but inflammatory angina, with a tendency to the formation of an abscess. This implies that only a certain and not too high a degree of inflammation is necessary to establish *croup;* that a higher degree is necessary to establish subsequent exudation—*diphtheritis;* but that the latter must not necessarily follow the former process, but that it can.

The condition of the mucous tissues in certain portions of the pharynx favors the development of the diphtheritic process. The looser it adheres to the muscles underneath, the more submucous tissue is interposed between them, the easier can an exudation accumulate and remain within its interstitial cavities. The mucous membrane of the pharynx, down to the œsophagus, shows, particularly in the neighborhood of the lymphatic glands, a very loose texture. Next to it stands, in looseness of texture, that of the tonsils, the velum palati, and the soft palate. In the larynx, we meet with a corresponding condition of the mucous membrane in the plicis ary-epiglotticus and between the two arytenoid cartilages, and on the rima glottidis posterior. In these localities, we must expect to meet diphtheritis.

Dr. Hoffman then shows successfully, from the literature of these two diseases, that his pathologico-anatomical conclusions correspond well with the observations of practical writers, as many reported cases show either croup or croup followed by diphtheritis; that, in some cases, the exudation could be peeled off at the commencement without trouble, and without injury to the mucous membrane (croup), but that this could not be accomplished later without injury to the mucous membrane (diphtheritis); that the localities marked out above as particularly favorable to diphtheritis are those where the disease has been observed to principally occur; and that diphtheritis and croup occur simultaneously in the same individual—diphtheritis in the pharynx, croup in the larynx—sometimes, diphtheritis in the pharynx and epiglottis, and croup in the larynx.

Examination of the croupous exudation under the microscope shows no trace of organization—an amorphous mass, with a few solitary blood-corpuscles, very few pus-corpuscles, and a few cell-like formations. Exudations from the mouth always show fungoid formations, while those from the larynx only show them when examined a few days after the patient's death.

To a long but quite interesting deviation from the subject, on the formation of pus-corpuscles, occasioned by the question how the cell-like bodies and pus-corpuscles, which are discoverable in the lowest strata of the infiltrated mucous membrane, originate, we can bestow but a very limited notice, as a decision of the question has not yet been arrived at. The only settled points are*: First, that the pus-corpuscles and the white blood-corpuscles are identical and the same, and that they originate from the same source—the lymph and the chyle; second, that the red corpuscles are developed within the blood, out of the white corpuscles.*

Finally, Hoffman returns to the original subject, and con tends that croup and diphtheritis, anatomically and pathologically considered, are only occasioned by the changes which the exudation and the surrounding tissues frequently undergo in diphtheritis. The exudation becomes subject to chemical decomposition, forces the enclosed tissues into necrotic decay, and leads to the formation of diphtheritic ulcers, and all clinical differences between the two diseases depend upon this process and its consequences.

It is well known how easily an infection of the blood can be accomplished by absorption from a very minute hearth (point of infection), and positive results have lately been obtained by actual experiment on this subject at Vienna.

It has been asserted that diphtheritis is a contagious infection of the blood, which occurs epidemically. *Bartels* has

---

*Erb. Contributions to the History of the Development of the Red Corpuscles. Virchow's Archiv., Vol. xxxiv., p. 138.

proven that epidemic and sporadic croup are not different from each other. The swelling of the lymphatic glands of the neck has been considered peculiar to diphtheritis; but it appears whenever ulcerations occur in the mouth, in the one disease as well as the other, and it is only more constant in diphtheritis because these ulcerations occur more constantly in that disease. The paralytic phenomena sometimes connected with diphtheria cannot constitute it a peculiar disease. They are the consequence of nutritive derangements of the nerve-extremities, caused by the pressure of the coagulated exudation. The changes in the spleen and the swelling of the intestinal follicles are caused by secondary infection of the blood, and are always observed where an infection of the blood takes place from an unhealthy ulcerated surface. Only after this blood-infection, the clinical image of diphtheritis becomes different from that of croup.

The origin of diphtheritis has frequently been ascribed to a species of fungus, and it has, on this account, been considered a specific disease. The mere presence of these cryptogamic organisms furnishes no tenable reason for this doctrine. Where have these fungi not been discovered? But even if they were the cause of the croupous-diphtheritic process, it would not effect any change in the anatomical process which the mucous membrane undergoes during the development and course of the disease.

---

*Diphtheritis and Diphtheria. By Dr. L. Letzerich, Idstein, near Weisbaden.*

As a continuation of former labors on diphtheritis, published in Vols. 45, 46 and 47 of Virchow's Archiv., Dr. Letzerich communicates in the February number of Vol. 52 of that journal the result of one hundred and eighteen additional experiments in relation to the origin of diphtheritis, and a series of interesting observations concerning the artificial production of secondary morbid phenomena of diphtheritis in rabbits, by feeding them on the fungus to which he attributes the origin of that disease.

He holds that this cryptogamic organism, a low form of fungus which enters and penetrates the mucous membranes from their exterior surface, causes the local diphtheritic exudation and the subsequent destruction of the corresponding portions of the mucous membrane of the tonsils, uvula, velum palati, etc. He experimented largely and produced by one and the same species of fungus always the same local symptoms.

Primarily, he considers diphtheritis a strictly local disease, which frequently appears without marked general morbid phenomena. In some instances the accompanying angina of the tonsils and pharynx can be connected with more or less fever. Constipation, pain in the bowels and headache frequently appear at the commencement. After a few alvine evacuations, and, generally, after the first removal of the diphtheritic coats, these symptoms disappear or improve. If these exudations are not carefully removed, fungi enter the stomach and intestinal canal. If this takes place, or if, by a destruction of the mucous membrane, the spores of this fungus enter deeply into the submucous tissue, the nature of the symptoms and the image of the disease in general undergo a very marked change. The patients become quite feverish, the pulse frequent and small, convulsions occur frequently, and a state of collapse, generally more or less interrupted by deceptive cheerfulness on the part of the patient, often causes sudden death. Where this collapse assumes any duration, vomiting, sometimes diarrhœa and intense abdominal pains, and flatulency accompany the same. A regular order in the development of the symptoms does not occur. Collapse or sopor, however, at least in a light degree, takes place in all cases.

The secretion of urine is generally diminished, and the urine contains, frequently, albumen. Under the microscope it exhibits granular cylinder and kidney epithelium, sometimes the latter alone, always more or less, but generally a considerable quantity of spores and tufts of fungi of the same species observed in the exudations. These tufts are always imbedded within a fine-grained, light yellow colored mass of exudation, which swims about in the urine and imparts to it a slightly turbid appearance.

For the production, by experiment, of the principal secondary symptoms, which have been mentioned above as the consequences of diphtheritic infection, Dr. Letzerich used a large number of rabbits, from six to eight weeks old—some older and some nearly full-grown. The infection was accomplished by fungi, raised by him from spores,* not by diphtheritic exudations. Some of these fungi were nearly two years old. Small pieces of light-bread, covered by these fungi, were rolled up into small pieces of the size of a grain of wheat, and given to these animals as food. The surface of these small pieces was carefully smoothed to prevent infection of the pharynx, which object was only partly accomplished, as small diphtheritic patches made their appearance on the lips and gums of several animals. The young animals ceased to eat thirty-six hours, the older ones forty-eight to fifty-two hours, after having swallowed the fungi; all became quiet—ceased to run about. Fluids, such as milk and water, were greedily swallowed by them. The temperature of their bodies increased considerably, and the smooth-haired animals assumed an ugly, grizzly appearance. They finally ceased to take fluids, and either diarrhœa or constipation, with almost entire cessation of the secretion of urine, set in. The development of these symptoms occupied less time in the younger animals than in the older ones. The younger ones were killed seventy-two hours, the older ones four days after the first infection, by severing the medulla oblongata.

On examination of these animals after death, the principal pathological changes consisted in the appearance of a large quantity of white granular masses protruding over the surface of the mucous membrane, from the size of a small shot to that of an English pea. They were observed on the mucous membrane of the stomach of *all* the young animals, and while they were less numerous generally, and entirely absent in the stomach of many of the older animals, they made their appearance in them almost invariably in the ileum. Similar appearances were present in the upper segment of the duode-

---

* Beiträge zur Therapic der Diphtheritis. Berliner Klinische Wochenschrift, 1869, No. 23.

num. Removed with the plate of a knife, these masses showed a grumous, but, in their particles, adherent substance. Wherever they had been located on the intestinal mucous membrane, ulcerations in the stomach, with sharp, irregular edges, often involving the whole thickness of the membrane, in the ileum only soft excavations, were found beneath them. Kidneys hyperæmic, bladders containing very little urine, which now and then contained albumen.

The stomach, upper part of the duodenum and kidneys of the younger, the ileum and kidneys of the older animals, were submitted to microscopic examination, the results of which in general were as follows:

The white masses consisted of the separated elements of mucous membrane, cells, filaments and a compact granular exudat, the whole favoring a mass of detritus, with a large quantity of fungus filaments, ripe and unripe spores. The extreme ends of the villi of the ileum were often destroyed, in consequence of which a communication was established between the receptacles of the chyle and the exudates containing the fungi. Spores and fragments of the same were plainly perceptible within them and, lower down, in the substance of Lieberkühn's glands, and generally in the deeper tissues of the mucous membrane.

The tubuli contorti of the cortical substance of the kidneys contained bright, shining spores; the tubuli recti of the medullary substance, extensive tufts of fungi in large quantities. On the surface of the papillæ facing the calices, a fine granular exudat, containing extensive tufts, prevailed. These same tufts were found swimming in the urine of the calices, ureters and bladders, in the same manner in which they had been observed in the urine of diphtheritic children, where deep ulcerations in the pharynx existed. In the tissues of the renal capsules of these rabbits extensive fungus-tufts were found, partly in groups, partly isolated, which tufts were always imbedded in a fine granular exudat, as was always the case in the kidneys, wherever fungi were found.

Dr. Letzerich concludes, from these experiments, that during the destruction of the mucous tissues an opening of the blood, chyle and lymph vessels is occasioned, through which fragments of fungi enter the general circulation and cause a grave general disease, with a serious disturbance of the functions of the kidneys. In this organ, the formation of new fungi is so enormous that spores and tufts are continually excreted in large quantities with the remarkably diminished quantity of urine.

The very same process occurs in the pharyngeal diphtheritis of children. The fungi bore themselves into the mucous membrane, cause openings in the lymph and blood-vessels, and a transition of fragments of fungi into the general circulation. During a longer or shorter duration of the local affection, all at once the image of a grave general lesion makes its appearance in consequence of this occurrence. The children become comatose, and collapse very suddenly. A considerable fever sets in, in some cases, and in others again we see convulsions. Constipation or diarrhœa, vomiting, and in all cases a marked diminution of the urinary secretion, takes place. The urine, microscopically examined, shows, as above described in the rabbits, the spores and tufts of the diphtheritic-fungus, kidney epithelium, and now and then granular epithelium.

Under proper treatment, consisting in tepid baths, aqua distil. and aqua calcis, of equal parts, and chinin hydrochlor. internally, a gradual decrease of the described symptoms takes place. The urinary secretion increases, often very considerably, and within two to four weeks a cure is established in most of the cases.

Whether the swallowing of diphtheritic membranes by children produces the same changes in the intestinal canal as those observed in rabbits, Dr. Letzerich is not able to state definitely, as he has not had an opportunity to make post-mortem examinations. He believes, however, judging from the fact that the phenomena of these changes—constipation, diarrhœa, flatulency, vomiting, etc.—occur in the children as they do in the rabbits, that the opinion is fully justifiable. He thinks it proper to distinguish between diphtheritis and diphtheria—designating by the first the local primary affection, by the second the general lesion which follows the transition of the fungi into the general circulation.

